# Supplementary material for: Prediction of ferroelectricity-driven Berry curvature enabling charge- and spin-controllable photocurrent in tin telluride monolayers
Source: Nat Commun. 2019 Sep 3;10:3965. doi: 10.1038/s41467-019-11964-6 (PMC6722129; doi:10.1038/s41467-019-11964-6)
Supplement: Supplementary file 1 — Supplementary Information [file 41467_2019_11964_MOESM1_ESM.pdf]

Supplementary Information for "Prediction of ferroelectricity-driven Berry curvature enabling charge- and spin-controllable photocurrent in tin telluride monolayers"

J. Kim *et al*.

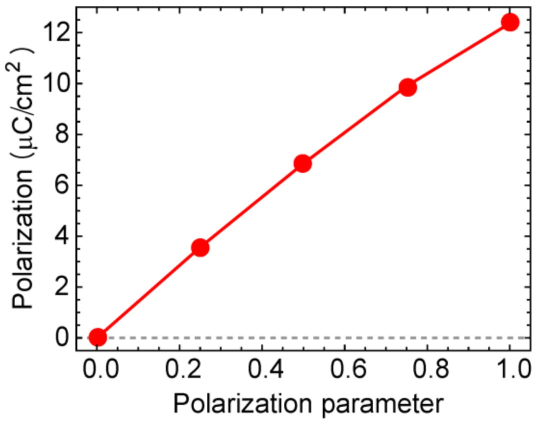

Supplementary Figure 1 | Evolution of spontaneous electric polarization. The calculated electric polarization of the SnTe monolayer is increased with the polarization parameter normalized by the native value along the *x*-axis.

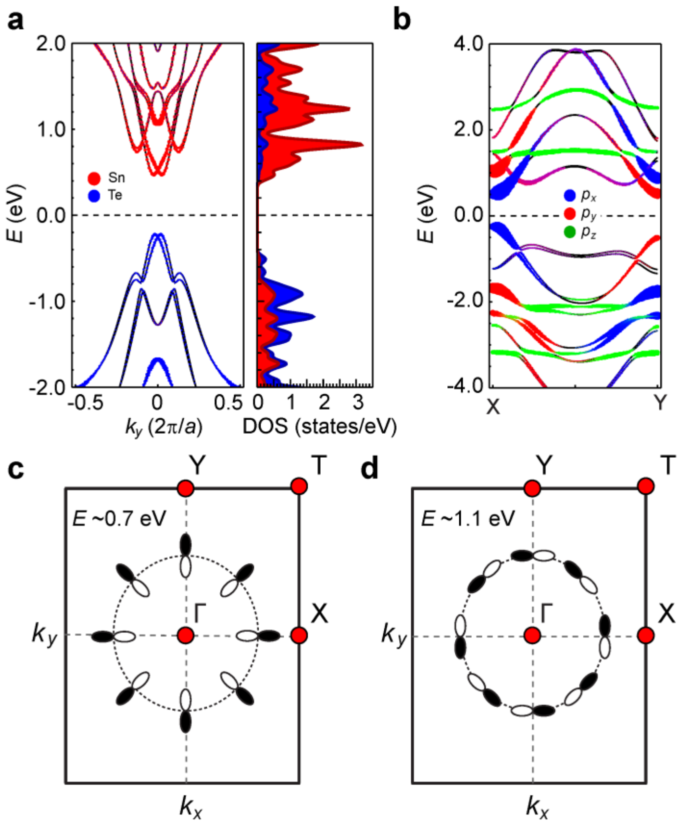

Supplementary Figure 2 | Electronic structure of SnTe. a, The atom-projected band structure and density of states (DOS) near the X valley. The contribution of Sn (Te) is represented in red (blue). **b**, The orbital-projected band structure along the circular path from the X valley to the Y valley. The contributions of p_x, p_y, and p_z orbitals are represented in blue, red, and green, respectively. **c-d,** The atomic orbital projection of (**c**) the lowest (~0.7

eV) and (**d**) the next lowest (~1.1 eV) conduction bands along the circular line. Special points (Γ, X, Y, T) are denoted by red dots.

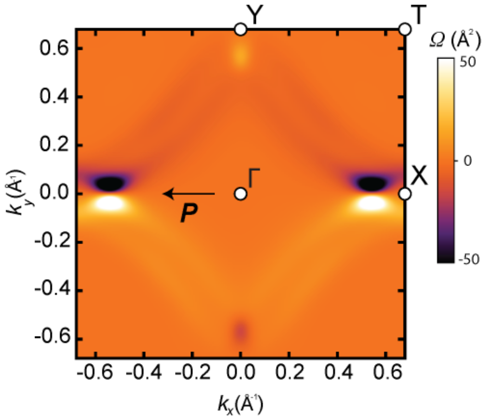

Supplementary Figure 3 | Berry curvature (*Ω*) of the SnTe monolayer with the opposite polarization direction. The **c**alculated Berry curvature (BC) map in the first Brillouin zone. The BC dipoles are formed at the X valleys.

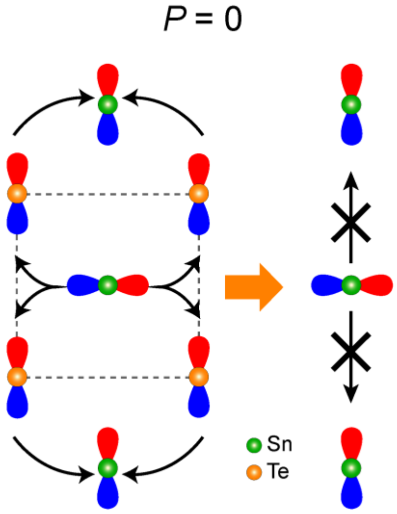

Supplementary Figure 4 | Schematic drawing of the inter-orbital hopping channel in the SnTe monolayer. The inter-orbital hopping between Sn atoms is absence without ferroelectric polarization. The dumbbells represent p_x and p_y orbitals of Sn and Te atoms; the blue (red) coloured region means a positive (negative) value of the *p* orbital wave function.

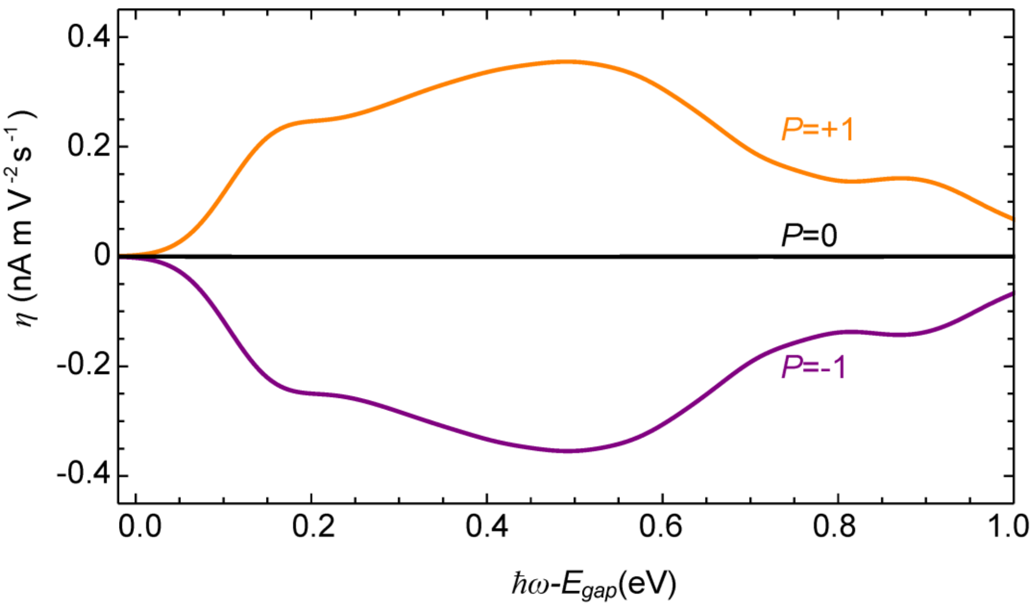

Supplementary Figure 5 | Calculated spectral dependence of the photocurrent in the SnTe monolayer. We calculate η by using the formalism in Ref. 2. The photocurrent is then determined by $dJ_y/dt = \eta E_x(\omega)E_y(-\omega)$. Note that the photocurrent is reversed by the polarization switching from *P*=+1 to *P*=-1. Due to the reduced dimensionality of the SnTe monolayer, the units of J and η are changed accordingly in consideration of the 10 Å thickness of the vacuum layer.

Supplementary Note 1 | Tight-binding derivation of the orbital Rashba effect. From the atomic structure of the SnTe monolayer as shown in Fig. 1a, we utilize the gliding symmetry of the system in constructing the tight-binding model. The system is invariant under the gliding operators $T(a\mathbf{x}')\nu_x$ and $T(a\mathbf{y}')\nu_x$, where T is the translational operator, $\mathbf{x}' = (\mathbf{x}+\mathbf{y})/\sqrt{2}$, $\mathbf{y}' = (-\mathbf{x}+\mathbf{y})/\sqrt{2}$, and ν_i (for $i = x, y, z$) is the Pauli matrix in Sn/Te space. The simultaneous eigenstates of these operators are

$$|\mathbf{k}, \mathrm{A}, \pm\rangle = \frac{1}{\sqrt{N}} \sum_{nm} e^{i\left(nk_{x'}+mk_{y'}\right)a} (\pm)^{n+m} |n, m, \mathrm{A}\rangle, \tag{1}$$

where $|n, m, \mathrm{A}\rangle$ is the electronic state localized at A=Sn, Te atom at the position $na\mathbf{x}' + ma\mathbf{y}'$, $|\mathbf{k}, \mathrm{A}, \pm\rangle$ is the corresponding Bloch state, and N is the total number of sites. Here $\pm$ degree of freedom comes from different eigenvalues for the gliding operators. Each Bloch state has 4 degrees of freedom: 2 for Sn/Te atomic sites and 2 for p_x/p_y-orbitals. For simplicity, we discard the spin degree of freedom, but the derivation keeps the same for spin cases. Below we write the Bloch state in terms of the momentum in the x, y coordinate, by using $k_{x,y} = \left(k_{x'} \mp k_{y'}\right)/\sqrt{2}$ and $a' = \sqrt{2}a$.

We note that $|\mathbf{k}, \mathrm{A}, +\rangle = |\mathbf{k} + (2\pi/a')\mathbf{y}, \mathrm{A}, -\rangle$, and thus the construction of the Hamiltonian only for + is sufficient for our purpose since the result for – is straightforwardly given by replacing $\mathbf{k}$ by $\mathbf{k} + (2\pi/a')\mathbf{y}$. The final result does not change with this choice.

As considering the nearest-neighbour hopping integrals, the hopping within a unit cell is given by $H_{\mathrm{hop},1} = -t_\pi \sum_{nm} \boldsymbol{c}_{n,m}^\dagger \nu_x \boldsymbol{c}_{n,m}$ because the hopping to the z direction is always composed of π-bonding. Here $\boldsymbol{c}_{n,m}^\dagger = \left(c_{n,m,\mathrm{Sn},p_x}^\dagger, c_{n,m,\mathrm{Sn},p_y}^\dagger, c_{n,m,\mathrm{Te},p_x}^\dagger, c_{n,m,\mathrm{Te},p_y}^\dagger\right)$, $\boldsymbol{c}_{n,m}$ is its conjugate transpose, and $c_{n,m,\mathrm{A},p_i}^\dagger$ is the creation operator for electrons localized at A atom at the site (n, m). We define the creation operators for the Bloch states $c_{\mathbf{k},\mathrm{A},p_i}^\dagger$ similarly.

The hopping processes to neighbouring unit cells are more complicated. If the two atoms are distant along $\mathbf{r}_1$ direction, the hopping integral of p_{r_1} orbitals is t_σ while the hopping integral of p_{r_2} is t_π where $\mathbf{r}_2 = \mathbf{z} \times \mathbf{r}_1$. Then the hopping term is given in the form of $t_\sigma |p_{r_1}\rangle\langle p_{r_1}| + t_\pi |p_{r_2}\rangle\langle p_{r_2}|$. Defining $\theta_\mathbf{v} = \arg(v_x + iv_y)$ for an arbitrary vector $\mathbf{v}$, $|p_{r_1}\rangle = \cos\theta_{\mathbf{r}_1}|p_x\rangle + \sin\theta_{\mathbf{r}_1}|p_y\rangle$ and $|p_{r_2}\rangle = \cos\theta_{\mathbf{r}_2}|p_x\rangle + \sin\theta_{\mathbf{r}_2}|p_y\rangle$. Then, the hopping term is written in $(|p_x\rangle, |p_y\rangle)$ basis and corresponds to the following matrix.

$$R(\theta_{\hat{\mathbf{r}}_1})\begin{pmatrix} t_\sigma & 0 \\ 0 & t_\pi \end{pmatrix} R(-\theta_{\hat{\mathbf{r}}_1}), \tag{2}$$

where $R(\theta)$ is the rotational matrix. For instance, the hopping term from $|n, m, \mathrm{Sn}\rangle$ to $|n+1, m, \mathrm{Te}\rangle$ in Fig. 3a is

$$\begin{pmatrix} c^\dagger_{n,m,\mathrm{Te},p_x} & c^\dagger_{n,m,\mathrm{Te},p_y} \end{pmatrix} R(\theta_{a\mathrm{x}'-d\mathrm{x}})\begin{pmatrix} t_\sigma & 0 \\ 0 & t_\pi \end{pmatrix} R(-\theta_{a\hat{\mathrm{x}}'-d\hat{\mathrm{x}}})\begin{pmatrix} c_{n,m,\mathrm{Sn},px} \\ c_{n,m,\mathrm{Sn},py} \end{pmatrix}, \tag{3}$$

where d is the atomic displacement of Sn atoms, which is the ferroelectric polarization parameter. One realizes that, for $d = 0$, $\theta_{a\mathrm{x}'-d\mathrm{x}} = \pi/4$ thus the hopping integrals for both p_x and p_y orbitals are both $(t_\sigma + t_\pi)/2$. However, for $d \neq 0$, the hopping integrals are different (Fig. 3a and Supplementary Fig. 4). We denote the collection of the all possible hopping terms by $H_{\mathrm{hop},2}$ without presenting its complicated form here. The periodicity of the system guarantees that $H_{\mathrm{hop}} = H_{\mathrm{hop},1} + H_{\mathrm{hop},2}$ is diagonal in $\mathbf{k}$. Note also that the magnitude of the hopping integrals are different for hoppings to the *x* direction and those to the –*x* direction due to different distances (see the difference between t'_{xy} and t_{xy} in Fig. 3a). We denote the difference hopping integrals by $t_{\sigma/\pi}$ and $t'_{\sigma/\pi}$ accordingly. Their difference is proportional to the ferroelectric polarization parameter d, and thus we define $t_{\sigma/\pi} - t'_{\sigma/\pi} \equiv d t_{\sigma/\pi,\Delta}$.

We take the simplest on-site term given by

$$H_{\text{on-site}}(\mathbf{k}) = \sum_{\substack{i=x,y \\ \text{A}=\text{Sn,Te}}} E_{\text{A}}(\mathbf{k}) c^{\dagger}_{\mathbf{k},\text{A},p_i} c_{\mathbf{k},\text{A},p_i}. \tag{4}$$

It is also possible to generalize this model. For instance, one may include spin-orbit coupling (SOC) to obtain $\mathbf{k}$-dependent SOC parameter introduced in the main text. After tedious algebra, we obtain the following total Hamiltonian $H_{\text{on-site}} + H_{\text{hop}}$.

$$H(\mathbf{k}) = \sum_{\substack{i=x,y \\ \text{A}=\text{Sn,Te}}} c^{\dagger}_{\mathbf{k},\text{A},p_i} h(\mathbf{k}) c_{\mathbf{k},\text{A},p_i} \tag{5}$$

$$\begin{aligned} h(\mathbf{k}) = &\begin{pmatrix} E_{\text{Sn}}(\mathbf{k}) & 0 \\ 0 & E_{\text{Te}}(\mathbf{k}) \end{pmatrix} \\ &- \left[t_\pi + 2(t_\sigma + t_\pi)\cos\frac{k_x a}{\sqrt{2}}\cos\frac{k_y a}{\sqrt{2}} - 2(t_\sigma - t_\pi)\tau_x \sin\frac{k_x a}{\sqrt{2}}\sin\frac{k_y a}{\sqrt{2}} \right] \nu_x \\ &- \left[2d(t_{\sigma,\Delta} + t_{\pi,\Delta})\sin\frac{k_x a}{\sqrt{2}}\cos\frac{k_y a}{\sqrt{2}} + 2d(t_{\sigma,\Delta} - t_{\pi,\Delta})\tau_x \cos\frac{k_x a}{\sqrt{2}}\sin\frac{k_y a}{\sqrt{2}} \right. \\ &\qquad \left. - \frac{2\sqrt{2}d}{a}(t_\sigma - t_\pi)\sin\frac{k_x a}{\sqrt{2}}\cos\frac{k_y a}{\sqrt{2}}\tau_z \right] \nu_y. \end{aligned} \tag{6}$$

The total Hamiltonian is not diagonal in Sn/Te space. To obtain an effective Hamiltonian for Sn only, we take the Schrieffer-Wolff transformation: $H' = e^S(H_{\text{on-site}} + H_{\text{hop}})e^{-S}$, where S is an anti-hermitian operator. In our case, the choice $S = \nu_z H_{\text{hop}}/[E_{\text{Sn}}(\mathbf{k}) - E_{\text{Te}}(\mathbf{k})]$ satisfies $[H_{\text{on-site}}, S] = H_{\text{hop}}$. Then, the Baker-Hausdorff lemma gives $H' = H_{\text{on-site}} + H_{\text{hop,eff}}$ where $H_{\text{hop,eff}} = [S, H_{\text{hop}}]/2$ is a second order correction of hoppings. Projecting $H_{\text{hop,eff}}$ to Sn gives an effective Hamiltonian for Sn. We denote the resulting 2×2 matrix by $H_{\text{hop,eff,Sn}}$.

After a long algebra, the τ_y component of $H_{\text{hop,eff,Sn}}$ up to first order in d is

$$\frac{1}{2}\text{Tr}[\tau_y H_{\text{hop,eff,Sn}}] = \frac{8d}{a'}\frac{(t_\sigma - t_\pi)^2}{E_{\text{Sn}}(\mathbf{k}) - E_{\text{Te}}(\mathbf{k})}\sin^2\frac{k_x a'}{2}\sin k_y a'. \tag{7}$$

Near the X valley, $k_x \approx k_{\text{X}}$ is mostly constant, thus we obtain the orbital Rashba term proportional to the ferroelectric parameter d.

To consider the SOC energy in the on-site term, one may add

$$H_{\text{on-site,SOC}}(\mathbf{k}) = \sum_{\text{A=Sn,Te}} \lambda_{\text{A}} \begin{pmatrix} c^{\dagger}_{\mathbf{k},\text{A},p_x} & c^{\dagger}_{\mathbf{k},\text{A},p_y} \end{pmatrix} \tau_y \sigma_z \begin{pmatrix} c_{\mathbf{k},\text{A},p_x} \\ c_{\mathbf{k},\text{A},p_x} \end{pmatrix}. \tag{8}$$

By applying the same Schrieffer-Wolff transformation, we obtain the following SOC term:

$$-\left\{\frac{8(t_\sigma - t_\pi)^2 \bar{\lambda}}{(E_{\text{Sn}} - E_{\text{Te}})^2} \sin^2\frac{k_x a'}{2} \sin^2\frac{k_y a'}{2} + \frac{2\Delta\lambda}{(E_{\text{Sn}} - E_{\text{Te}})^2}\left[t_\pi + 2(t_\sigma + t_\pi)\cos\frac{k_x a'}{2}\cos\frac{k_y a'}{2}\right]^2\right\}\tau_y\sigma_z, \tag{9}$$

which contribute to $H_{\text{hop,eff,Sn}}$ as an effective SOC energy in the form of $H_{\text{SOC}}(\mathbf{k}) = \lambda_{\mathbf{k}}\tau_y\sigma_z$ as shown in the main text. Here $\bar{\lambda} = (\lambda_{\text{Sn}} + \lambda_{\text{Te}})/2$ and $\Delta\lambda = (\lambda_{\text{Sn}} - \lambda_{\text{Te}})/2$.

Supplementary Note 2 | Comparison between the analytic model and DFT calculations.

We start from the model Hamiltonian for the conduction Sn-p_{x} and Sn-p_{y} bands near the X valley.

$$H(\mathbf{k}) = H_0(\mathbf{k}) + H_{\text{SOC}}(\mathbf{k}) + H_{\text{FE}}(\mathbf{k}), \tag{10}$$

$$H_0(\mathbf{k}) = E_{\text{X}}(\mathbf{k}) - J\cos 2\theta_{\mathbf{k}}\,\tau_z - J\sin 2\theta_{\mathbf{k}}\,\tau_x, \tag{11}$$

$$H_{\text{SOC}}(\mathbf{k}) = \frac{2\lambda_{\mathbf{k}}}{\hbar^2}\mathbf{L}\cdot\mathbf{S}, \tag{12}$$

$$H_{\text{FE}}(\mathbf{k}) = \alpha_L k_y L_z = |\alpha_L|\mathbf{L}\cdot(\hat{\mathbf{P}}\times\mathbf{k}). \tag{13}$$

where, in addition to the other terms introduced in the main text, we introduce the SOC Hamiltonian $H_{\text{SOC}}(\mathbf{k})$ to reproduce also the Rashba type band structure presented in Fig. 1c. Here, $\lambda_{\mathbf{k}}$ is the effective SOC strength for Sn atoms and $\mathbf{S}$ is the spin angular momentum operator. The effective SOC contains the $\mathbf{k}$ dependence as we integrate out the Te p-orbital degrees of freedom as explicitly shown above. Within the p_{x}, p_{y}-orbital subspace, $H_{\text{SOC}}(\mathbf{k}) = \lambda_{\mathbf{k}}\sigma_z\tau_y$, where σ_i is the spin Pauli matrix.

Keeping the first-order terms in $\lambda_{\mathbf{k}}$, the energy eigenvalues of $H(\mathbf{k})$ are given by

$$E_{\mathbf{k}n\sigma} = E_{\text{X}}(\mathbf{k}) + (-1)^n\left(J_{\mathbf{k}} + \sigma\frac{\lambda_{\mathbf{k}}\alpha_L\hbar}{J_{\mathbf{k}}}k_y\right), \tag{14}$$

where $n(=1{,}2)$ and $\sigma(=\pm)$ are the orbital and spin indices, respectively, and $J_{\mathbf{k}} = \sqrt{J^2 + (\alpha_L \hbar k_y)^2}$ is the modified orbital splitting. The last term in Supplementary Eq. (14) represents a k_y-linear spin splitting, which is the unidirectional Rashba spin splitting that is shown in Fig. 1c.[33] The spin texture calculated from $\langle \psi_{n\sigma}(\mathbf{k})|\mathbf{S}|\psi_{n\sigma}(\mathbf{k})\rangle = \sigma(\hbar/2)\mathbf{z}$ also accords with our DFT calculations.

The BC of the lowest energy conduction band $(n = 1)$ is obtained as

$$\Omega(\mathbf{k}) = \frac{2\alpha_L J^2}{J_{\mathbf{k}}^3 \hbar} \partial_{k_x} \theta_{\mathbf{k}}, \tag{15}$$

which reproduces the main features of the BC dipole presented in Fig. 2a. From the linear dependence of the BC on the orbital Rashba coefficient α_L, $\Omega(\mathbf{k})$ is (i) induced by the ferroelectric polarization and (ii) switchable by reversing *P*. The BC is also (iii) independent of $\lambda_{\mathbf{k}}$ (up to first order), (iv) an odd function of k_y, and (v) an even function of k_x.

The orbital Rashba effect of Supplementary Eq. (13) leads to a finite expectation value of the *z*-component orbital angular momentum, which is zero without the ferroelectrically induced anti-symmetric hopping (Fig. 3a, b). The orbital angular momentum texture from our analytic model is written as

$$\langle \psi_{n\sigma}(\mathbf{k})|\mathbf{L}|\psi_{n\sigma}(\mathbf{k})\rangle = (-1)^n \left[\frac{\alpha_L \hbar^2}{J_{\mathbf{k}}} k_y + \sigma \frac{\lambda_{\mathbf{k}} \hbar}{J_{\mathbf{k}}}\left(1 - \frac{\alpha_L^2 \hbar^2}{J_{\mathbf{k}}^2} k_y^2\right)\right] \mathbf{z}. \tag{16}$$

The orbital angular momentum that is evaluated from the DFT calculation without SOC (Fig. 3c, d) is consistent with the first term in Supplementary Eq. (16); the orbital angular momentum texture (i) is odd in k_y (thus, it changes its sign at $k_y = 0$ within a single band), (ii) has the opposite signs for *n* =1 and 2, and (iii) changes its sign by reversing *P*. Based on the agreement with the DFT results, our minimal model demonstrates that the intriguing BC structure of the SnTe monolayer is developed via the orbital Rashba effect that originates

from the in-plane ferroelectricity.

As a side remark, we confirmed that extending our model by including all the p orbitals does not alter our results in Supplementary Eqs. (14)–(16), up to first order in $\lambda_{\mathbf{k}}$.

Supplementary Note 3 | Spin Berry curvature distribution. To support the validity of our minimal model presented in the main text, we additionally calculate the spin-resolved BC distribution and compare it with the DFT calculations.

Supplementary Fig. 6 shows the DFT calculation for the spin BC, given by

$$\Omega_s(\mathbf{k}) = -\frac{\hbar}{2}\mathrm{Im}\sum_{n}\sum_{n'\neq n} f_n \frac{\langle\psi_n(\mathbf{k})|\{\sigma_z, v_x\}|\psi_{n'}(\mathbf{k})\rangle\langle\psi_{n'}(\mathbf{k})|v_y|\psi_n(\mathbf{k})\rangle}{(E_{n'}(\mathbf{k}) - E_n(\mathbf{k}))^2}. \quad (17)$$

Similar to the BC distribution, the spin BC is mainly concentrated near the X valley. Different from the BC whose total sum over the Brillouin zone is vanishing due to the time-reversal symmetry, the spin BC gives a non-zero net flux. This implies that a large spin Hall conductivity can be acquired when the SnTe monolayer is slightly doped.

A distinction between Ω and Ω_s can also be found in their dependence on ferroelectricity and SOC. While the BC varies in proportion to P, the spin BC remains large irrespective of P (Supplementary Fig. 6b). Moreover, the overall sign of the spin BC is independent of the polarization direction. Meanwhile, the spin BC drastically changes with varying λ (Supplementary Fig. 6c); in contrast to the BC, the spin BC scales with λ and disappears as λ goes to zero (Supplementary Fig. 6d). Our results indicate that the SOC is the key ingredient for the spin BC while the ferroelectricity is less relevant.

The symmetry argument presented in the main text can also be applicable to the spin BC, $\Omega_s(\mathbf{k}) = \Omega_+(\mathbf{k}) - \Omega_-(\mathbf{k})$. The time reversal symmetry and the mirror symmetry of the system imply $\Omega_s(\mathbf{k}) = \Omega_s(-\mathbf{k})$ and $\Omega_s(k_x, k_y) = \Omega_s(k_x, -k_y)$ respectively. Without SOC, the two spin channels become identical and thus $\Omega_+(\mathbf{k}) = \Omega_-(\mathbf{k})$. Consequently,

$\Omega_s(\mathbf{k})$ is zero without λ (Supplementary Fig. 6d). Once we consider the ferroelectric reversal, $\Omega_\pm^{+P}(\mathbf{k}) = \Omega_\pm^{-P}(-\mathbf{k})$ implies $\Omega_s^{+P}(\mathbf{k}) = \Omega_s^{-P}(\mathbf{k})$ (Supplementary Fig. 6e). Therefore, the symmetry argument well explains the DFT results shown in Supplementary Fig. 6a-c.

Lastly, we compare the DFT calculation with our analytic model. From the minimal model presented in the main text, we obtain

$$\Omega_s(\mathbf{k}) = \frac{J^2}{J_{\mathbf{k}}^3 \hbar}(\nabla_{\mathbf{k}}\theta_{\mathbf{k}} \times \nabla_{\mathbf{k}}\lambda_{\mathbf{k}})_z - \frac{3\hbar\lambda_{\mathbf{k}}\alpha_L^2 J^2 k_y}{J_{\mathbf{k}}^5}\partial_{k_x}\theta_{\mathbf{k}}, \tag{18}$$

which reproduces the spin BC distribution presented in Supplementary Fig. 6 well: (i)

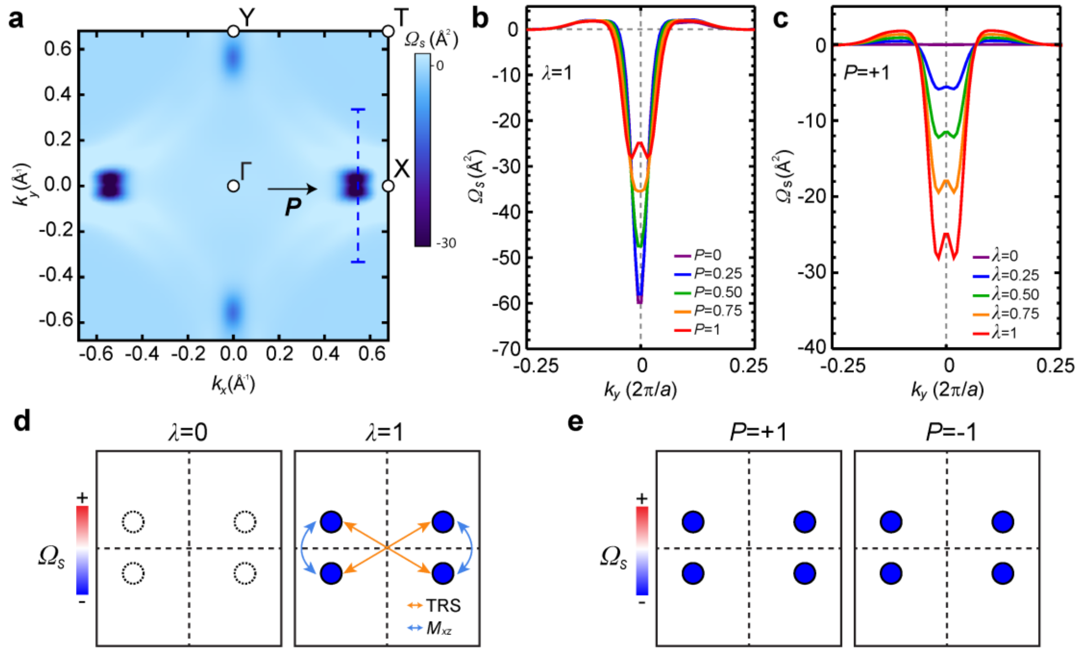

Supplementary Figure 6 | spin Berry curvature (Ω_s) of SnTe monolayer. a, Calculated spin Berry curvature (BC) map in the first Brillouin zone. The spin BC monopole is formed at the X valleys. **b-c,** the spin BC calculated along the vertical blue line in (**a**) for varying the magnitude of (**b**) the ferroelectric polarization (P) and (**c**) the SOC strength (λ), where the values of P and λ are normalized by the native values. **d,** Schematic drawings for the spin BC following the symmetry of the system. **e,** Schematic drawings for the response of the spin BC to the ferroelectric switching.

dependent on $\lambda_{\mathbf{k}}$, (ii) independent of the sign of P, and (iii) an even function of k_x and k_y. The first term in Supplementary Eq. (18) is independent of the ferroelectric polarization (up to first order), which explains the non-vanishing $\Omega_{\mathrm{S}}(\mathbf{k})$ at $P = 0$ ($\alpha_L = 0$) in Supplementary Fig. 6b. The second term is the ferroelectric contribution in the spin BC, originating from both the orbital Rashba effect and the SOC.

Supplementary Note 4 | Calculation of the optoelectronic responses using the analytic theory. To analytically calculate the optoelectronic responses, we start with the Hamiltonian in Supplementary Eq. (10) and calculate the charge and spin photogalvanic effect.

According to Ref. 1, the charge current generated by the right/left-circularly polarized light is given by

$$J_{y,\pm} = \pm\frac{2\pi e^3\tau E_0^2}{\hbar^2}D^{\mathrm{inter}}(\omega) = \frac{e^3\tau E_0^2}{\pi\hbar^2}\int \Theta(\hbar\omega - \Delta E(\mathbf{k}))\partial_{k_y}\Omega_{vc,\pm}(\mathbf{k})\, d^2k,$$
$$\Omega_{vc,\pm}(\mathbf{k}) = \sum_{v,c}\frac{\left|\langle v|\partial_{k_x}H \pm i\partial_{k_y}H|c\rangle\right|^2}{2[\Delta E(\mathbf{k})]^2}, \qquad (19)$$

where $\pm$ refers to the right and left circularly polarized light, respectively, τ is the momentum relaxation time, and E_0 is the field amplitude of the light.

To calculate $\Omega_{vc,\pm}(\mathbf{k})$, we consider electrons near X valley ($k_x \approx k_X$ and $k_y \approx 0$) and keep first order deviations. Since the Hamiltonian is spin diagonal, we approximate $|c\rangle = |\mathrm{Sn},\sigma\rangle, |v\rangle = |\mathrm{Te},\sigma\rangle$ and sum the contributions for each spin $\sigma = \pm$. For the orbital part, we may approximate $\langle v|\boldsymbol{\tau}|v\rangle \approx (0, -\alpha_L\hbar k_y/J, -1)$ for the highest-valance band, and $\langle c|\boldsymbol{\tau}|c\rangle = -\langle v|\boldsymbol{\tau}|v\rangle$ for the lowest conduction band. With these projections, $\Delta E(\mathbf{k}) = E_{\mathrm{Te}}(\mathbf{k}) - E_{\mathrm{Sn}}(\mathbf{k}) - 2\bar{\lambda}\sigma\alpha_L\hbar k_y/J$, where the last term is nothing but the spin Rashba effect. We neglect higher order contributions from the ferroelectric polarization. Lastly, we take a parabolic approximation $E_{\mathrm{Te}}(\mathbf{k}) \approx -\hbar^2\mathbf{k}^2/2m_{\mathrm{Te}}$ and $E_{\mathrm{Sn}}(\mathbf{k}) \approx E_{\mathrm{gap}} + \hbar^2\mathbf{k}^2/2m_{\mathrm{Sn}}$ thus $\Delta E(\mathbf{k}) \approx E_{\mathrm{gap}} + \hbar^2\mathbf{k}^2/2m_{vc}$, where $m_{vc}^{-1} = m_{\mathrm{Sn}}^{-1} + m_{\mathrm{Te}}^{-1}$. After some algebra,

$$D_y^{\mathrm{inter}}(\omega) = -d\frac{4m_{vc}a^2}{\pi\hbar^3\omega}\left[t_\sigma^2 - t_\pi^2 + a^{'}\left(t_\sigma t_{\pi,\Delta} + t_\pi t_{\sigma,\Delta}\right)\right]\left(1 - \frac{E_{\mathrm{gap}}}{\hbar\omega}\right)$$
$$\propto \alpha_L\left(1 - \frac{E_{\mathrm{gap}}}{\hbar\omega}\right), \quad (20)$$

$$J_{y,\pm} = \mp d\frac{16m_{vc}e^3\tau E_0^2a^2}{2\hbar^5\omega}\left[t_\sigma^2 - t_\pi^2 + a^{'}\left(t_\sigma t_{\pi,\Delta} + t_\pi t_{\sigma,\Delta}\right)\right]\left(1 - \frac{E_{\mathrm{gap}}}{\hbar\omega}\right)$$
$$\propto \alpha_L\left(1 - \frac{E_{\mathrm{gap}}}{\hbar\omega}\right). \quad (21)$$

Since the ferroelectric polarization P is proportional to d, we obtain the following rules.

$$J_{y,+}(P) = -J_{y,-}(P),$$
$$J_{y,\pm}(P) = -J_{y,\pm}(-P). \quad (22)$$

which is consistent with the charge photocurrent of Fig. 5g in the main text.

Next, we calculate the spin photogalvanic effect. The spin current is written as

$$J_{y,s,\pm} = \frac{e^3\tau E_0^2}{\pi\hbar^2}\int \Theta(\hbar\omega - \Delta E(\mathbf{k}))\partial_{k_y}\Omega_{vc,s,\pm}(\mathbf{k})\, d^2k,$$
$$\Omega_{vc,s,\pm}(\mathbf{k}) = \sum_{v,c}\frac{\langle v|\left\{\sigma_z, \partial_{k_x}H \pm i\partial_{k_y}H\right\}|c\rangle\langle c|\partial_{k_x}H \mp i\partial_{k_y}H|v\rangle}{4[\Delta E(\mathbf{k})]^2} \quad (23)$$
$$\approx \sum_{v,c}\sigma\frac{\left|\langle \mathrm{Te}, \sigma|\partial_{k_x}H \pm i\partial_{k_y}H|\mathrm{Sn}, \sigma\rangle\right|^2}{2[\Delta E(\mathbf{k})]^2}.$$

With the same approximation, we obtain the following spin current.

$$J_{y,s,\pm} = \alpha_L\frac{8m_{vc}\bar{\lambda}e^3\tau E_0^2a^2}{E_{\mathrm{gap}}^2J\hbar^5\omega^2}\left(t_\sigma^2 + t_\pi^2\right)$$
$$\times\left[\frac{3a^2\left(\hbar\omega - E_{\mathrm{gap}}\right)^2E_{\mathrm{gap}}m_{vc}}{\hbar^2}\cos^2\frac{k_Xa^{'}}{2}\right. \quad (24)$$
$$\left. + 2\left(\hbar^2\omega^2 - E_{\mathrm{gap}}^2\right)\sin^2\frac{k_Xa^{'}}{2}\right],$$

which implies

$$J_{y,s,+}(P) = J_{y,s,-}(P), \quad (25)$$

$$J_{y,s,\pm}(P) = -J_{y,s,\pm}(-P),$$

and fully consistent with the spin part of Fig. 5g in the main text.

Supplementary References

1 Xu, S.-Y. et al., Electrically switchable Berry curvature dipole in the monolayer topological insulator WTe_2. *Nat. Phys.* **14**, 900-906 (2018).

2 Sipe, J. E. & Shkrebtii, A. I. Second-order optical response in semiconductors. *Phys. Rev. B* **61**, 5337-5352 (2000).
